# The association of three promoter polymorphisms in *interleukin*-*10* gene with the risk for colorectal cancer and hepatocellular carcinoma: A meta-analysis

**DOI:** 10.1038/srep30809

**Published:** 2016-08-04

**Authors:** Yan-Hui Shi, Dong-Mei Zhao, Yue-Fei Wang, Xue Li, Man-Ru Ji, Dan-Na Jiang, Bai-Ping Xu, Li Zhou, Chang-Zhu Lu, Bin Wang

**Affiliations:** 1 Department of Gastroenterology, The First Hospital of Qiqihar City, Qiqihar, Heilongjiang, China; 2 Department of Physiology, Qiqihar Medical University, Qiqihar, Heilongjiang, China; 3 Intervention Therapy Department, The First Hospital of Qiqihar City, Qiqihar, Heilongjiang, China; 4 Central Laboratory, Qiqihar Medical University, Qiqihar, Heilongjiang, China

## Abstract

Mounting evidence supports a potent inhibitory role of interleukin-10 (IL-10) in tumor carcinogenesis, angiogenesis and metastasis. This meta-analysis was designed to examine the association of three promoter polymorphisms (−592C > A, −819C > T and −1082G > A) in *IL-10* gene with the risk for colorectal cancer and hepatocellular carcinoma. Qualification assessment and data collection were completed by two authors independently. The random-effects model using the DerSimonian and Laird method was fitted by the STATA software. Twenty-five articles involving 5933 cases and 9724 controls were meta-analyzed. Overall comparisons of the mutant alleles (−592A, −819T and −1082A) of three promoter polymorphisms with alternative wild alleles failed to reveal any statistical significance for both colorectal cancer and hepatocellular carcinoma (P > 0.05), and the likelihood of heterogeneity was low (*I*^2^ < 50%). For −592C > A polymorphism, a significant risk for colorectal cancer was identified when analysis was restricted to East Asians (odds ratio or OR = 1.41, 95% confidence interval or CI: 1.18–1.68, P < 0.001) and retrospective studies (OR = 1.23, 95% CI: 1.09–1.39, P = 0.001). As weighed by the Egger’s test and the fill-and-trim method, there was a low probability of publication bias for all studied polymorphisms. Our findings collectively suggest that the −592C > A polymorphism in *IL-10* gene might be a susceptibility locus for colorectal cancer in East Asians.

Interleukin-10 (IL-10) is an anti-inflammatory and immune-suppressive cytokine^1^,^2^. Mounting evidence supports a potent inhibitory role of IL-10 in tumor carcinogenesis, angiogenesis and metastasis^3^. Lack of IL-10 in turn can trigger the production of pro-inflammatory cytokines, prevent anti-tumor immunity and promote tumor growth^4^. In humans, IL-10 is encoded by *IL-10* gene on chromosome 1q31-q32 (gene ID: 3586), which comprises 5 exons and 4 introns. So far, there are 354 validated single nucleotide polymorphisms identified in *IL-10* gene (http://www.ncbi.nlm.nih.gov/gene/3586). Human IL-10 *in vivo* is produced mainly by T-cells, B-cells, monocytes and macrophages, and its changes are under strong genetic control, with an estimated heritability of as high as 75%^5^. In view of above evidence, it would be tempting to speculate that *IL-10* genetic alterations may contribute not only to circulating IL-10 variation but also to cancer susceptibility.

Of validated polymorphisms in *IL-10* gene, three promoter polymorphisms including −592C > A (rs1800872), −819C > T (rs1800871) and −1082G > A (rs1800896) are well-defined and have been widely evaluated in predisposition to cancer at some sites^6^,^7^,^8^,^9^,^10^. Many studies that tested whether the polymorphisms in the promoter region of *IL-10* gene are associated with hepatocellular carcinoma or colorectal cancer have shown controversial and inconclusive results^8^,^11^,^12^,^13^, at least in part because these studies are individually underpowered and involve different ethnic groups. Two previous meta-analyses have separately examined the association of these promoter polymorphisms with colorectal cancer and hepatocellular carcinoma^14^,^15^. Given accumulating data afterwards, we decided to conduct an updated meta-analysis on the association of three promoter polymorphisms in *IL-10* gene with the risk of having colorectal cancer and hepatocellular carcinoma among 5933 cases and 9724 controls from 25 articles published in English.

## Methods

### Checklist

To improve the quality of a systematic review, this meta-analysis was conducted according to the statement put forward by the Preferred Reporting Items for Systematic Reviews and Meta-Analyses (PRISMA)^16^.

### Search strategies

Potentially relevant articles were retrieved by searching Medline (PubMed), EMBASE (Excerpta Medica Database) and Web of Science using the following subject words: (interleukin-10 OR IL-10 OR IL 10) AND (colorectal cancer OR colon cancer OR rectal cancer OR hepatocellular carcinoma OR liver cancer) AND (allele OR genotype OR polymorphism OR variant OR mutation) as of January 1, 2016. All retrieved articles were managed by the EndNote X5 software (available at the website www.endnote.com, Thomson Reuters).

### Qualification assessment

As a prerequisite, all potential articles must be published in English. In addition, articles were qualified if they simultaneously satisfied the following criteria: (1) clinical endpoint: colorectal cancer or hepatocellular carcinoma; (2) study design: retrospective or nested case-control design; (3) studied polymorphisms: at least one of the three promoter polymorphisms, −592C > A, −819C > T and −1082G > A, in *IL-10* gene under investigation; (4) genetic data: the genotype or allele distributions of studied polymorphism(s) between cases and controls or the associated odds ratio (OR) and 95% confidence interval (CI). In case of duplicated publications from the same study group, article with a larger sample size was retained. Qualification assessment was completed independently by two investigators (Yan-Hui Shi and Chang-Zhu Lu), and if necessary a discussion was made over any uncertainties encountered.

### Information collection

From each qualified article, the same two investigators (Yan-Hui Shi and Chang-Zhu Lu) collected and typed relevant information into a standardized Excel template, including the first author’s surname, publication year, the country where study subjects resided, race, cancer type, matched condition, source of controls, study design, sample size, age, gender, smoking, drinking and family history of cancer, hepatitis B virus (HBV) and hepatitis C virus (HCV), as well as the genotype or allele distributions of studied polymorphisms between cases and controls. Two independently-completed templates were cross-checked with inconsistencies solved by consensus. The detailed characteristics of all qualified articles are summarized in Table 1.

### Statistical analysis

All statistical analyses are carried out with the STATA software for the Windows version 12.0 (StataCorp, College Station, Texas, USA). For all studied polymorphisms, deviation from the Hardy-Weinberg equilibrium for each polymorphism was assessed by the Chi-squared test or the Fisher’s exact test where appropriate in control groups at a significance level of 5%.

To statistically quantify the between-study heterogeneity, the inconsistency index (*I*^2^) is calculated, and it denotes the percent of observed diversity that is explained by heterogeneity rather than by chance. If the *I*^2^ is over 50% - a generally accepted cutoff value, it is indicative of significant heterogeneity. Individual effect-size estimates, ORs and its 95% CIs, were calculated under the fixed-effects model adopting the Mantel-Haenszel method^17^ when no significant heterogeneity was observed. Otherwise, the random-effects model adopting the DerSimonian and Laird method^18^ was used. In addition, to seek the clinical sources of heterogeneity, a set of stratified analyses by cancer type, race, matched condition, source of controls, study design and sample size were separately implemented. To avoid chance results, only subgroups involving 2 or more studies were analyzed. Moreover, a meta-regression analysis modeling age, gender, smoking, drinking and family history of cancer, HBV and HCV (HBV and HCV for hepatocellular carcinoma only) was conducted.

Influential analysis was conducted to see whether individual studies contribute significantly to pooled estimates by omitting each study one at a time sequentially.

Publication bias is a type of bias originating from the fact that studies with positive findings are more likely to be published than studies with negative findings, and its probability is weighted by the Egger’s linear regression test^19^ and the trim-and-fill method^20^. The trim-and-fill method is used to estimate the number of potential missing studies that might exist in a meta-analysis as presented by a filled funnel plot and the effect that these studies might have had on its effect-size estimate.

## Results

### Qualified articles

**Figure 1:**
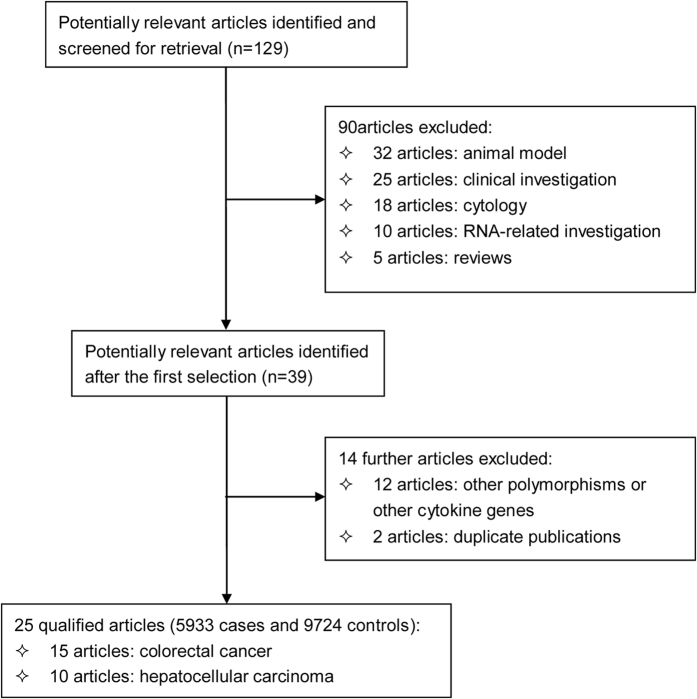
The selection process of all qualified articles in this meta-analysis.

The selection process of qualified articles is charted in Fig. 1. The initial retrieval identified a total of 129 potentially relevant articles using *ex-ante* subject words. Finally, only 25 articles passed pre-defined qualification assessment,^8^,^11^,^12^,^13^,^21^,^22^,^23^,^24^,^25^,^26^,^27^,^28^,^29^,^30^,^31^,^32^,^33^,^34^,^35^,^36^,^37^,^38^,^39^,^40^,^41^ and of them 15 used colorectal cancer (15 studies: 3938 cases and 6192 controls)^8^,^11^,^23^,^26^,^27^,^29^,^30^,^31^,^32^,^33^,^35^,^36^,^37^,^40^,^41^ and 10 used hepatocellular carcinoma (10 studies: 1995 cases and 3532 controls)^10^,^11^,^18^,^19^,^21^,^22^,^25^,^31^,^35^,^36^ as the clinical endpoint. For −592C > A, −819C > T and −1082G > A polymorphisms, there were respectively 11 and 7 studies, 3 and 5 studies, 11 and 6 studies for colorectal cancer and hepatocellular carcinoma.

### Baseline characteristics

Of 25 qualified studies, 12 were conducted in Caucasians, 10 in East Asians and 3 in mixed ethnicities. There were 14 studies having matched cases and controls, 3 studies unmatched and 8 studies unknown. Sixteen out of 25 studies enrolled controls from general populations and 9 from hospitals. Nineteen of 25 studies were retrospective case-control studies and 6 were nested case-control studies. TaqMan technique was the most widely adopted genotyping method (15 out of 25 studies). There were 17 studies with total sample size of 300 or more, and 8 studies of less than 300.

For both colorectal cancer and hepatocellular carcinoma, cases tended to be older (P = 0.022 and 0.028, respectively), male gender (P = 0.078 and 0.081, respectively) and smokers (P = 0.035 and 0.059) relative to controls. Moreover for hepatocellular carcinoma, the percentage of cases with HVB was exceedingly higher than that of controls (81.94% vs. 39.16%, P = 0.007).

### Overall estimates

**Figure 2:**
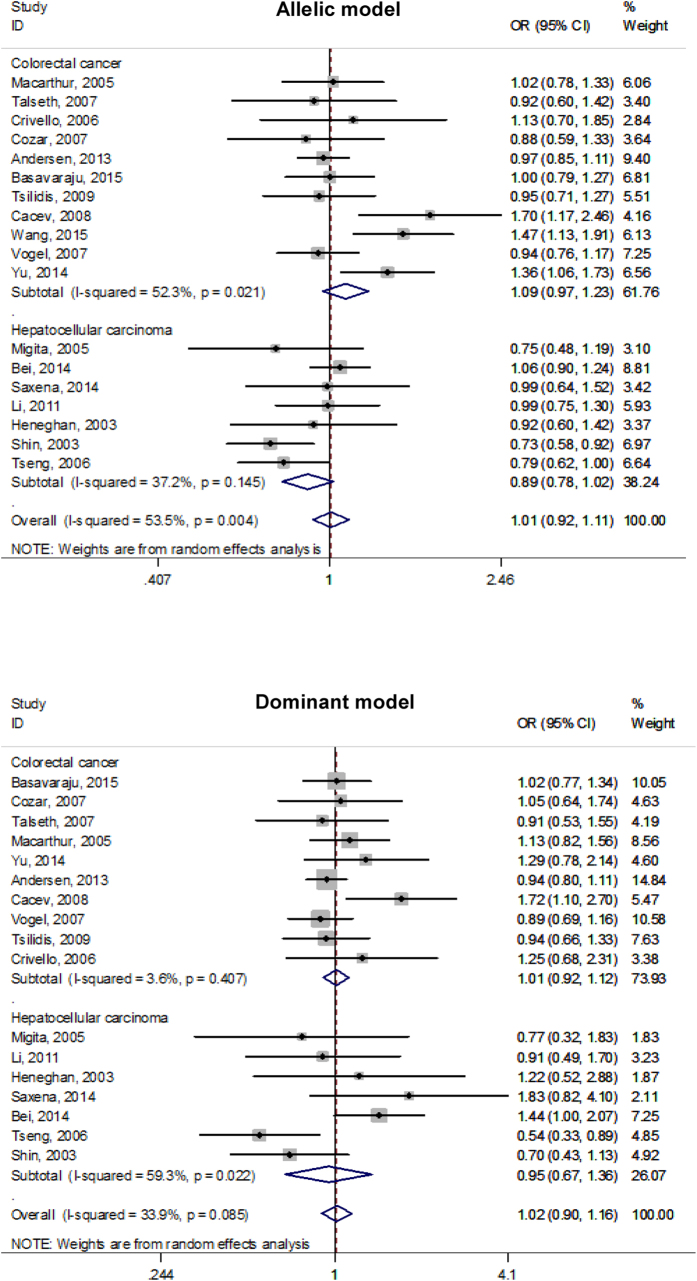
Forest plots of *IL-10* gene −592C > A polymorphism with colorectal cancer and hepatocellular carcinoma under both allelic and dominant models.

**Figure 3:**
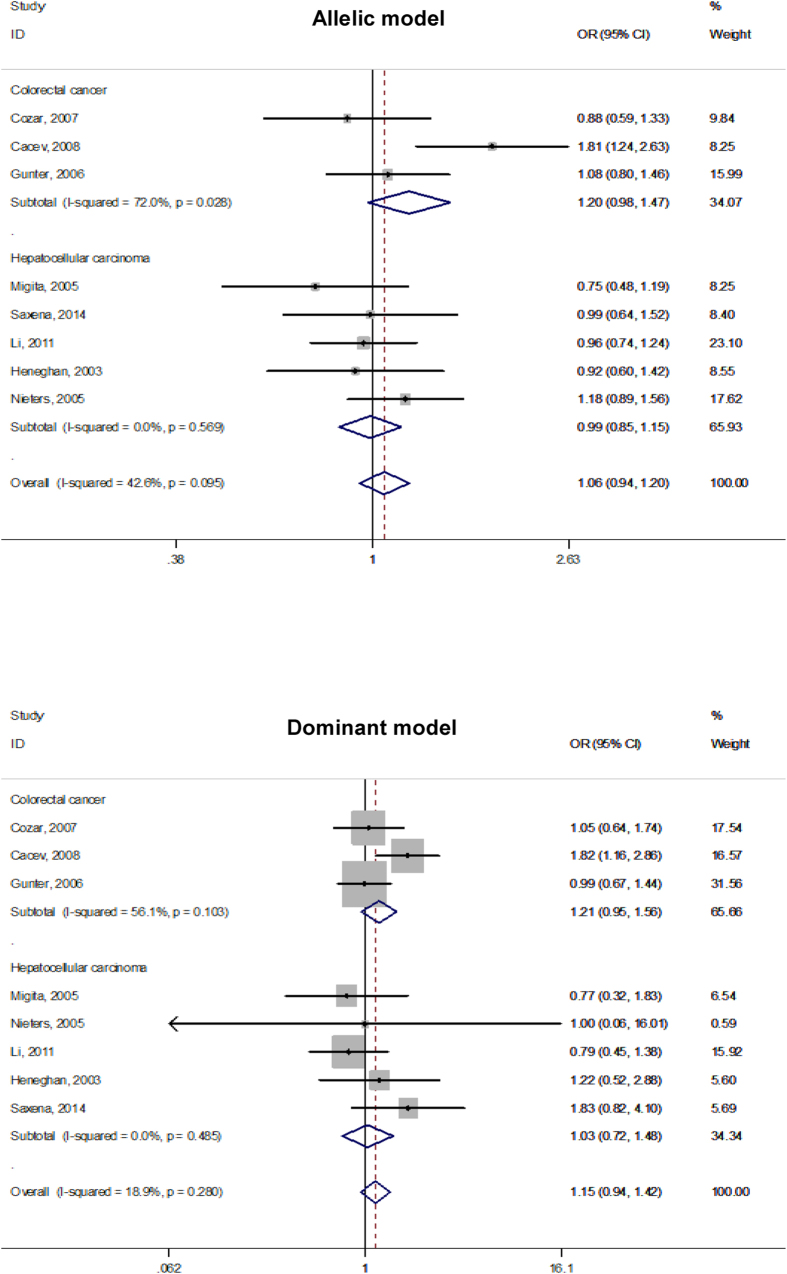
Forest plots of *IL-10* gene −819C > T polymorphism with colorectal cancer and hepatocellular carcinoma under both allelic and dominant models.

**Figure 4:**
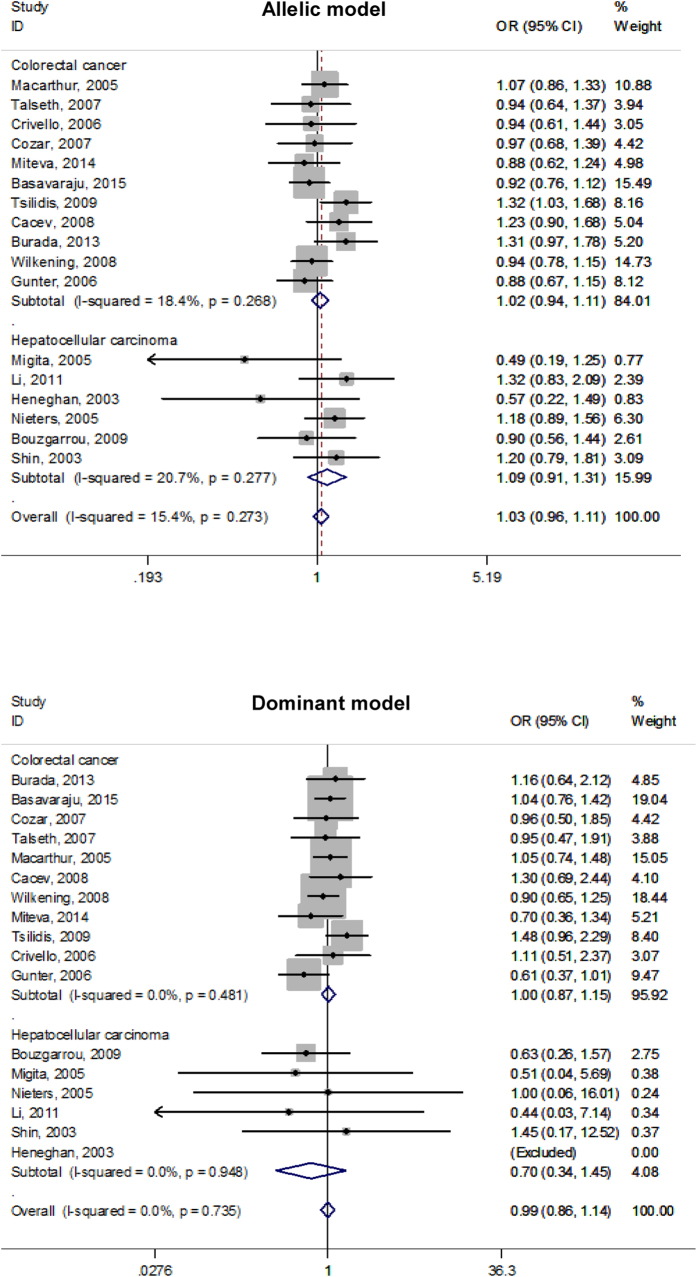
Forest plots of *IL-10* gene −1082G > A polymorphism with colorectal cancer and hepatocellular carcinoma under both allelic and dominant models.

Given the small number of mutant homozygous genotypes of three studied polymorphisms, individual effect-size estimates were pooled only on the basis of both allelic and dominant models. Overall comparisons of the mutant alleles (−592A, −819T and −1082A) with the alternative wild alleles failed to reveal any statistical significance (P > 0.05) for both colorectal cancer and hepatocellular carcinoma under both allelic and dominant models (Figs 2, 3, 4), and there was no indication of between-study heterogeneity as measured by the *I*^2^ (<50%), except for the association of −592C > A polymorphism with colorectal cancer under the allelic model (*I*^2^ = 52.3%) and with hepatocellular carcinoma under the dominant model (*I*^2^ = 59.3%), as well as for the association of −891C > T polymorphism with colorectal cancer under both allelic (*I*^2^ = 72.0%) and dominant (*I*^2^ = 56.1%) models.

### Stratified estimates

Considering the limited number of qualified studies for −819C > T polymorphism, the exploration of clinical heterogeneity by stratified analyses was only presented for −592C > A and −1082G > A polymorphisms under both allelic and dominant models (Tables 2 and 3). For −592C > A polymorphism, a significant increased risk for colorectal cancer was identified when analysis was restricted to East Asians under the allelic model (OR = 1.41, 95% CI: 1.18–1.68, P < 0.001) and to retrospective studies under both allelic (OR = 1.23, 95% CI: 1.09–1.39, P = 0.001) and dominant (OR = 1.21, 95% CI: 1.00–1.45, P = 0.047) models, and there was no evidence of significant heterogeneity. In contrast to hepatocellular carcinoma, there was no observable significance, except for a marginally significant association between −592C > A polymorphism and hepatocellular carcinoma in retrospective studies under the allelic model (OR = 0.90, 95% CI: 0.81–1.00, P = 0.051) and in studied with matched cases and controls under the dominant model (OR = 1.40, 95% CI: 1.00–1.97; P = 0.048). In addition, no statistical significance was noted in the other subgroups for −592C > A polymorphism and in all subgroups for −1082G > A polymorphism (P > 0.05).

### Influential analysis

For three studied polymorphisms in *IL-10* gene associated with colorectal cancer and hepatocellular carcinoma, influential analyses confirmed the overall changes in direction and magnitude under both allelic and dominant models.

### Meta-regression analysis

By modeling age, gender, smoking, drinking and family history of cancer, HBV and HCV (HBV and HCV for hepatocellular carcinoma only), the meta-regression analyses failed to detect any positive signals for three studied polymorphisms in association with both colorectal cancer and hepatocellular carcinoma under both allelic and dominant models (Supplementary Table S1).

### Publication bias

**Figure 5:**
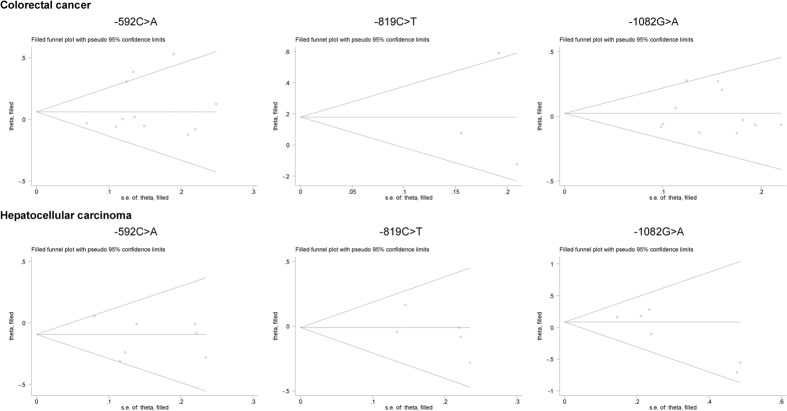
Filled funnel plots of the −592C > A, −819C > T and −1082G > A polymorphisms in IL-10 gene with colorectal cancer and hepatocellular carcinoma under the allelic model.

**Figure 6:**
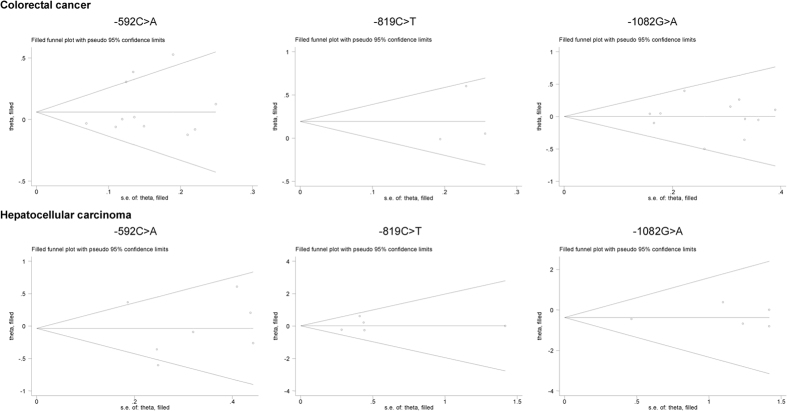
Filled funnel plots of the −592C > A, −819C > T and −1082G > A polymorphisms in IL-10 gene with colorectal cancer and hepatocellular carcinoma under the dominant model.

As weighed by the Egger’s test, there was a low probability of publication bias for three studied polymorphisms, except for −1082G > A polymorphism in association with hepatocellular carcinoma under the allelic model (Egger’s test: P = 0.042). As estimated by the trim-and-fill method, no missing studies were required to make the filled funnel plots symmetrical for three studied polymorphisms under both allelic (Fig. 5) and dominant (Fig. 6) models.

## Discussion

Through a comprehensive meta-analysis of three promoter polymorphisms in *IL-10* gene with colorectal cancer and hepatocellular carcinoma, we found that the −592C > A polymorphism might be a susceptibility locus for colorectal cancer in East Asians. Besides ethnic heterogeneity, study design might be another potential source of clinical heterogeneity for the association between −592C > A polymorphism and colorectal cancer. To our knowledge, this is so far the largest meta-analysis that has evaluated *IL-10* gene multiple promoter polymorphisms with colorectal cancer and hepatocellular carcinoma risk.

Differing from the findings of previous meta-analysis by Zhang *et al*. who enrolled subjects of only Caucasian descent^14^, we observed a significant association of −592C > A polymorphism with colorectal cancer in East Asians rather than in Caucasians. One possible reason for this failed confirmation in Caucasians might be the enlarged sample size, as the contrast of 3938 cases and 6192 controls in the current meta-analysis with 1469 cases and 2566 controls in the meta-analysis by Zhang *et al*.^14^. Another possible reason might be the confounding impact of source of controls since after restricting analysis to population-based studies, significance was detected in the meta-analysis by Zhang *et al*.^14^ but not in the current meta-analysis. However, a note of caution should be sounded for the significant association of −592C > A polymorphism with colorectal cancer in East Asians in this study since only two studies are available for analysis^11^,^41^ and a possible chance of publication bias cannot be excluded, albeit no evidence of between-study heterogeneity observed. A large-scale study in East Asian populations is thereby required to confirm this preliminary finding.

Through exhaustive data explorations, there is no hint of significance for the association of three studied polymorphisms in *IL-10* gene with hepatocellular carcinoma in this meta-analysis, inconsistent with the findings of the previous meta-analysis by Wei *et al*.^15^, as they observed a susceptible role of −592C > A polymorphism in hepatocellular carcinogenesis by pooling individual effect-size estimates of four Asian populations. In contrast to the 7 East Asian populations^12^,^21^,^22^,^24^,^25^,^28^,^38^,^39^ in this meta-analysis, our findings didn’t lend any credence to this susceptible role. Besides the enhanced statistical power in this meta-analysis, it might be the confounding impact of unaccounted heterogeneity in East Asians (*I*^2^ = 37.2% in contrast to 0.0% in Wei *et al*’s meta-analysis^15^). Moreover, as a corroboration of our negative findings, the association magnitude between −592C > A polymorphism and hepatocellular carcinoma risk was identical between the small and the large studies in our stratified analysis. Nevertheless, in spite of the negative findings in this study, it does not mean that the three studied polymorphisms in *IL-10* gene are not biologically functional, and it is possible that the relative risk attributable to a single allele is small^42^. To yield statistically reliable evidence, further studies incorporating a wide range of candidate genes responsible for the development of hepatocellular carcinoma are required to get a clear picture of its underlying genetic architecture.

Finally, some possible limitations need to be acknowledged when interpreting and extrapolating our meta-analytical findings. First, our literature retrieval was only limited to articles published in English, and doing so might introduce a selection bias^43^. However, the Egger’s test and the filled funnel plots for three studied polymorphisms indicated no evidence of publication bias in this meta-analysis. Second, pooled analysis was only restricted to three promoter polymorphisms in *IL-10* gene, and the other polymorphisms were not considered due to insufficient available data. Third, the only significant finding in this meta-analysis was based only on two eligible studies, leaving some room for further criticism. Fourth, all enrolled studies are case-control in design, which precluded the causality exploration. Fifth, only the risk of having colorectal cancer or hepatocellular carcinoma was treated as the clinical endpoint, and it is of interest to investigate whether the studied polymorphisms are associated with the recurrence and survival during subsequent medical therapies.

Taken together, we in an updated meta-analysis of three promoter polymorphisms in *IL-10* gene found that the -592C > A polymorphism might be a susceptibility locus for colorectal cancer in East Asians. Considering the ubiquity of genetic heterogeneity and in view of small sample sizes involved, our findings should be considered to be preliminary until being replicated or confirmed in other larger, well-designed studies in future investigations.

## Additional Information

**How to cite this article**: Shi, Y.-H. *et al*. The association of three promoter polymorphisms in *interleukin-10* genewith the risk for colorectal cancer and hepatocellular carcinoma: A meta-analysis. *Sci. Rep.* **6**, 30809; doi: 10.1038/srep30809 (2016).

## Supplementary Material

Supplementary Information

## Figures and Tables

**Table 1:** The detailed characteristics of all qualified studies in this meta-analysis.

Author, year	Cancer type	Ethnicity	Match	Source of controls	Study design	Genotyping	Sample size		Age (yrs)		Male (%)	
							Cases	Controls	Cases	Controls	Cases	Controls
Heneghan, 2003	HCC	East Asian	YES	Population	Retrospective	Probe	98	97	55.00	55.00	92.86	92.86
Shin, 2003	HCC	East Asian	NA	Hospital	Retrospective	Single-base extension	230	792	55.80	48.40	NA	NA
Macarthur, 2005	CRC	Caucasian	YES	Population	Retrospective	TaqMan	264	408	NA	NA	56.80	51.50
Migita, 2005	HCC	East Asian	NO	Hospital	Retrospective	Sequencing	48	188	62.50	51.50	81.25	67.55
Nieters, 2005	HCC	East Asian	YES	Hospital	Retrospective	Allele-specific method	250	250	49.30	49.30	88.00	88.00
Crivello, 2006	CRC	Caucasian	YES	Population	Retrospective	Allele-specific method	62	124	NA	NA	NA	NA
Gunter, 2006	CRC	Mixed	YES	Hospital	Retrospective	TaqMan	244	231	60.00	57.00	77.50	63.60
Tseng, 2006	HCC	East Asian	NA	Hospital	Retrospective	Chip	208	528	55.00	51.50	NA	NA
Cozar, 2007	CRC	Caucasian	NA	Population	Retrospective	TaqMan	96	176	68.15	59.00	66.70	66.70
Talseth, 2007	CRC	Caucasian	NA	Hospital	Retrospective	Probe	118	110	NA	NA	NA	NA
Vogel, 2007	CRC	Caucasian	YES	Population	Nested	TaqMan	355	753	59.00	56.00	56.34	55.51
Cacev, 2008	CRC	Caucasian	NA	Population	Retrospective	TaqMan	160	160	64.50	63.10	53.10	53.70
Wilkening, 2008	CRC	Caucasian	YES	Population	Nested	TaqMan	308	585	56.80	56.80	43.50	43.90
Bouzgarrou, 2009	HCC	Caucasian	YES	Population	Retrospective	Allele-specific method	58	103	61.60	46.00	34.48	40.78
Ognjanovic, 2009	HCC	Mixed	YES	Population	Retrospective	TaqMan	120	230	60.50	59.50	68.33	60.43
Tsilidis, 2009	CRC	Caucasian	YES	Population	Nested	TaqMan	208	381	62.80	62.80	46.10	45.40
Li, 2011	HCC	East Asian	NO	Population	Nested	SNPlex assay	204	415	NA	NA	77.90	69.20
Andersen, 2013	CRC	Caucasian	NA	Population	Nested	KASP assay	970	1789	58.00	56.00	56.39	53.33
Burada, 2013	CRC	Caucasian	YES	Hospital	Retrospective	TaqMan	144	233	65.91	63.69	60.42	61.37
Bei, 2014	HCC	East Asian	YES	Hospital	Retrospective	TaqMan	720	784	48.65	47.72	87.18	83.29
Miteva, 2014	CRC	Mixed	YES	Population	Retrospective	ARMS	119	154	65.52	65.52	59.66	NA
Saxena, 2014	HCC	East Asian	NO	Hospital	Retrospective	RFLP	59	145	55.31	35.50	94.91	76.52
Yu, 2014	CRC	East Asian	YES	Population	Retrospective	RFLP	299	296	62.27	61.72	52.84	52.70
Basavaraju, 2015	CRC	Caucasian	NA	Population	Nested	TaqMan	388	496	64.00	62.00	67.00	55.20
Wang, 2015	CRC	East Asian	NA	Population	Retrospective	MassArray	203	296	NA	NA	NA	NA
	Smoking (%)		Drinking (%)			Family history (%)			HBV (%)		HCV (%)	
Author, year	Cases	Controls	Cases	Controls		Cases	Controls		Cases	Controls	Cases	Controls
Heneghan, 2003	NA	NA	NA	NA		NA	NA		83.00	0.00	NA	NA
Shin, 2003	NA	NA	NA	NA		NA	NA		100.00	100.00	NA	NA
Macarthur, 2005	54.40	53.50	79.60	80.30		NA	NA		NA	NA	NA	NA
Migita, 2005	NA	NA	NA	NA		NA	NA		100.00	100.00	0.00	0.00
Nieters, 2005	45.20	34.40	36.80	16.00		12.00	0.80		82.00	14.00	3.60	1.20
Crivello, 2006	NA	NA	NA	NA		NA	NA		NA	NA	NA	NA
Gunter, 2006	11.10	4.80	NA	NA		16.80	11.90		NA	NA	NA	NA
Tseng, 2006	NA	NA	NA	NA		NA	NA		100.00	65.15	NA	NA
Cozar, 2007	NA	NA	NA	NA		NA	NA		NA	NA	NA	NA
Talseth, 2007	NA	NA	NA	NA		NA	NA		NA	NA	NA	NA
Vogel, 2007	69.30	65.47	NA	NA		NA	NA		NA	NA	NA	NA
Cacev, 2008	NA	NA	NA	NA		0.00	0.00		NA	NA	NA	NA
Wilkening, 2008	NA	NA	NA	NA		NA	NA		NA	NA	NA	NA
Bouzgarrou, 2009	NA	NA	NA	NA		NA	NA		NA	NA	100.00	0.00
Ognjanovic, 2009	41.67	28.26	70.83	62.61		NA	NA		29.20	11.70	48.30	0.40
Tsilidis, 2009	51.40	47.20	NA	NA		12.20	7.20		NA	NA	NA	NA
Li, 2011	NA	NA	39.58	32.28		26.11	9.42		64.70	24.58	8.95	2.89
Andersen, 2013	70.52	66.24	NA	NA		NA	NA		NA	NA	NA	NA
Burada, 2013	NA	NA	NA	NA		NA	NA		NA	NA	NA	NA
Bei, 2014	38.89	14.67	40.00	14.41		NA	NA		78.60	37.00	NA	NA
Miteva, 2014	NA	NA	NA	NA		NA	NA		NA	NA	NA	NA
Saxena, 2014	NA	NA	NA	NA		NA	NA		100.00	0.00	NA	NA
Yu, 2014	35.12	37.84	27.42	25.68		NA	NA		NA	NA	NA	NA
Basavaraju, 2015	55.90	50.90	86.60	75.80		18.6	15.2		NA	NA	NA	NA
Wang, 2015	NA	NA	NA	NA		NA	NA		NA	NA	NA	NA

*Abbreviations*: HCC, hepatocellular carcinoma; CRC, colorectal cancer; NA, not available; HBV, hepatitis B virus; HCV, hepatitis C virus.

**Table 2:** Stratified analyses of the −592C > A and −1082G > A polymorphisms in *IL-10* gene with colorectal cancer and hepatocellular carcinoma risk under the allelic model.

Subgroup	−592C > A polymorphism						Subgroup	−1082G > A polymorphism				
	Num. of studies	OR	95% CI	P	*I*^2^			Num. of studies	OR	95% CI	P	*I*^2^
*Colorectal cancer*												
Ethnicity	East Asian	2	1.41	1.18–1.68	<0.001	0.0%	East Asian	9	1.05	0.96–1.15	0.278	20.0%
	Caucasian	9	1.00	0.92–1.09	0.993	12.5%	Mixed	2	0.88	0.71–1.09	0.237	0.0%
Matched	YES	5	1.06	0.94–1.20	0.340	31.1%	YES	7	1.04	0.94–1.15	0.407	36.4%
Source	Population	10	1.10	0.97–1.25	0.134	56.2%	Population	8	1.02	0.94–1.12	0.621	16.1%
	Hospital	1	—*	—	—	—	Hospital	3	1.02	0.86–1.22	0.802	48.9%
Study design	Retrospective	7	1.23	1.09–1.39	0.001	47.8%	Retrospective	8	1.03	0.92–1.15	0.596	0.0%
	Nested	4	0.97	0.88–1.07	0.498	0.0%	Nested	3	1.03	0.84–1.27	0.749	65.9%
HWE	YES	10	1.03	0.95–1.12	0430	38.1%	YES	9	1.03	0.94–1.12	0.586	18.2%
Sample size	<300	3	0.96	0.74–1.24	0.745	0.0%	<300	4	0.93	0.77–1.12	0.432	0.0%
	≥300	8	1.12	0.97–1.29	0.118	64.4%	≥300	7	1.05	0.96–1.14	0.323	44.5%
*Hepatocellular carcinoma*												
Ethnicity	East Asian	7	0.91	0.83–1.01	0.065	37.2%	East Asian	5	1.13	0.93–1.38	0.226	28.1%
	Caucasian	0	—	—	—	—	Caucasian	1	—			
Matched	YES	2	1.04	0.90–1.21	0.602	0.0%	YES	3	1.05	0.83–1.33	0.680	22.4%
	NO	3	0.94	0.76–1.15	0.545	0.0%	NO	2	0.88	0.34–2.29	0.792	71.1%
Source	Population	2	0.97	0.77–1.22	0.794	0.0%	Population	3	1.03	0.76–1.39	0.872	30.9%
	Hospital	5	0.86	0.72–1.04	0.115	56.2%	Hospital	3	1.13	0.90–1.42	0.290	37.2%
Study design	Retrospective	6	0.90	0.81–1.00	0.051	45.3%	Retrospective	5	1.05	0.86–1.28	0.618	27.0%
	Nested	1	—	—	—	—	Nested	1	—	—	—	—
HWE	YES	4	0.85	0.68–1.04	0.117	66.4%	YES	4	1.08	0.85–1.39	0.517	7.6%
Sample size	<300	3	0.89	0.69–1.15	0.365	0.0%	<300	3	0.76	0.52–1.12	0.163	0.0%
	≥300	4	0.89	0.74–1.07	0.215	65.7%	≥300	3	1.21	0.98–1.49	0.072	0.0%

*Abbreviations*: OR, odds ratio; 95% CI, 95% confidence interval; HWE, Hardy-Weinberg equilibrium. P value was calculated under the random-effects model adopting the DerSimonian and Laird method. *Data are not shown due to the limited number of qualified articles (n < 2).

**Table 3:** Stratified analyses of the −592C > A and −1082G > A polymorphisms in *IL-10* gene with colorectal cancer and hepatocellular carcinoma risk under the dominant model.

		−592C > A polymorphism					Subgroup	−1082G > A polymorphism				
Subgroup		Num. of studies	OR	95% CI	P	*I*^2^		Num. of studies	OR	95% CI	P	*I*^2^
*Colorectal cancer*												
Ethnicity	East Asian	1	—*	—	—	—	Caucasian	9	1.07	0.92–1.24	0.378	0.0%
	Caucasian	9	1.00	0.91–1.11	0.986	4.5%	Mixed	2	0.64	0.43–0.95	0.028	0.0%
Matched	YES	5	1.02	0.87–1.19	0.841	0.0%	YES	7	0.98	0.83–1.16	0.821	31.4%
Source	Population	9	1.02	0.92–1.12	0.775	12.7%	Population	8	1.05	0.90–1.22	0.575	0.0%
	Hospital	1	—	—	—	—	Hospital	3	0.83	0.59–1.16	0.268	28.7%
Study design	Retrospective	6	1.21	1.00–1.45	0.047	0.0%	Retrospective	8	0.95	0.78–1.15	0.583	0.0%
	Nested	4	0.94	0.84–1.06	0.323	0.0%	Nested	3	1.07	0.87–1.30	0.531	38.3%
HWE	YES	10	1.01	0.92–1.12	0.830	3.6%	YES	9	1.04	0.89–1.20	0.655	0.0%
Sample size	<300	3	1.05	0.77–1.43	0.776	0.0%	<300	4	0.90	0.64–1.27	0.553	0.0%
	≥300	7	1.01	0.91–1.12	0.895	30.7%	≥300	7	1.03	0.88–1.19	0.751	26.6%
*Hepatocellular carcinoma*												
Ethnicity	East Asian	7	0.95	0.67–1.36	0.794	59.3%	East Asian	5	0.85	0.25–2.85	0.786	0.0%
	Caucasian	0	—	—	—	—	Caucasian	1	—	—	—	—
Matched	YES	2	1.40	1.00–1.97	0.048	0.0%	YES	3	0.66	0.28–1.57	0.350	0.0%
	NO	3	1.09	0.71–1.67	0.696	21.9%	NO	2	0.48	0.08–2.95	0.425	0.0%
Source	Population	2	1.01	0.61–1.68	0.969	0.0%	Population	3	0.61	0.26–1.45	0.266	0.0%
	Hospital	5	0.93	0.58–1.49	0.774	72.3%	Hospital	3	0.98	0.25–3.86	0.977	0.0%
Study design	Retrospective	6	0.96	0.64–1.46	0.864	66.0%	Retrospective	5	0.73	0.34–1.54	0.402	0.0%
	Nested	1	—	—	—	—	Nested	1	—	—	—	—
HWE	YES	4	0.82	0.50–1.36	0.449	74.3%	YES	4	0.70	0.32–1.55	0.381	0.0%
Sample size	<300	3	1.25	0.77–2.03	0.364	4.6%	<300	3	0.62	0.26–1.45	0.268	0.0%
	≥300	4	0.85	0.53–1.36	0.504	73.9%	≥300	3	0.98	0.24–4.00	0.980	0.0%

*Abbreviations*: OR, odds ratio; 95% CI, 95% confidence interval; HWE, Hardy-Weinberg equilibrium. P value was calculated under the random-effects model adopting the DerSimonian and Laird method. *Data are not shown due to the limited number of qualified articles (n < 2).
