# Supplementary material for: The association of three promoter polymorphisms in interleukin-10 gene with the risk for colorectal cancer and hepatocellular carcinoma: A meta-analysis
Source: Sci Rep. 2016 Aug 4;6:30809. doi: 10.1038/srep30809 (PMC4973248; doi:10.1038/srep30809)
Supplement: Supplementary Information [file srep30809-s1.pdf]

The association of three promoter polymorphisms in *interleukin-10* gene with the risk for colorectal cancer and hepatocellular carcinoma: A meta-analysis

Short title: *IL-10 and the Risk for CRC and HCC*

Yan-Hui Shi[2], Dong-Mei Zhao[2], Yue-Fei Wang[1], Xue Li[1], Man-Ru Ji[2], Dan-Na Jiang[2], Bai-Ping Xu[3], Li Zhou[4], Chang-Zhu Lu[1,#], Bin Wang[1,#]

Author affiliations:

[1]Department of Physiology, Qiqihar Medical University, Qiqihar, Heilongjiang, China; [2]Department of Gastroenterology and [3]Intervention Therapy Department, The First Hospital of Qiqihar City, Qiqihar, Heilongjiang, China; [4]Central Laboratory, Qiqihar Medical University, Qiqihar, Heilongjiang, China.

[#]Correspondence and reprints should be addressed to:

Chang-Zhu Lu, M.D., Ph.D. or Bin Wang, M.D. Ph.D.

Address: Bukui North Street No. 333, Jianhua District, Qiqihar city 161006, Heilongjiang province, China.

Tel and Fax: +86-452-2663183.

E-mail: changzhulu1998@163.com (CZL) or wangbinqqhr@163.com (BW).

Supplementary Table S1. Summary of meta-regression analysis of baseline characteristics for three examined polymorphisms in *IL-10* gene under both allelic and dominant models

Characteristics	Allelic model (P value)			Dominant model (P value)		
	**-592C>A**	**-819C>T**	**-1082G>A**	**-592C>A**	**-819C>T**	**-1082G>A**
Colorectal cancer						
Age	0.318	0.466	0.183	0.997	0.382	0.965
Gender	0.352	0.567	0.214	0.861	0.496	0.948
Smoking	0.987	–*	0.545	0.561	–	0.549
Drinking	0.726	–	–	0.542	–	–
Family cancer history	0.954	–	0.921	0.971	–	0.864
Hepatocellular carcinoma						
Age	0.329	0.305	0.727	0.782	0.57	0.436
Gender	0.327	0.304	0.893	0.140	0.809	0.349
Smoking	–	–	–	–	–	–
Drinking	–	–	–	–	–	–
Family cancer history	–	–	–	–	–	–
HBV	0.159	0.660	0.501	0.448	0.922	0.823
HCV	–	0.941	0.867	–	0.497	0.388

Abbreviations: HBV, hepatitis B virus; HCV, hepatitis C virus. *P value is not available due to insufficient observations.
